# Supplementary material for: Deep reinforcement learning for optimal experimental design in biology
Source: PLoS Comput Biol. 2022 Nov 21;18(11):e1010695. doi: 10.1371/journal.pcbi.1010695 (PMC9721483; doi:10.1371/journal.pcbi.1010695)
Supplement: S1 Text — (PDF) [file pcbi.1010695.s001.pdf]

1 Fitted Q-learning can design optimal experiments on a simple Monod growth system

To test the potential of using reinforcement learning for OED, we first applied the Fitted Q-learning algorithm to a simple non-linear system with Monod dynamics to investigate whether the agent is capable of optimising the D-optimality score with the given information. We compare the performance of the FQ agent with a one step ahead optimiser (OSAO). In this system we have one state variable, x, and no measurement noise so that output $\mathbf{Y} = x$. There is one control variable u that is controlled by the OSAO or the RL controller. The dynamics of the system are given by a simple Monod relationship between $\frac{dx}{dt}$ and u:

$$\frac{dx}{dt} = \frac{p_1 u}{p_2 + u} x,$$

where p_1 and p_2 are the parameters to be estimated.

In the following $p_1 = p_2 = 1$. Both the RL and OSAO implementations start from initial condition $x_0 = 1$, $u_0 = 0.5$ and are able to choose u between $0 \leq u \leq 0.1$. The FQ agent works in a discrete action space and therefore has ten equally spaced discrete actions to choose from (distributed uniformly between 0 and 0.1). Fig AA shows the experimental input profiles chosen by the FQ agent and OSAO, which are similar. These consist of inputs at the highest level available and a value about half way between the maximum and minimum values. The FQ agent selects inputs values that straddle the optimum found by the OSAO, likely due to the discrete nature of its action space. Fig AB shows the system trajectories of both controllers. As expected these are very similar. Fig AC shows the performance of the FQ agent as it was trained for 500 episodes, compared to the performance of the trajectory found by the OSAO. Their performance is similar; by the end of training, the FQ agent is performing slightly better than the OSAO.

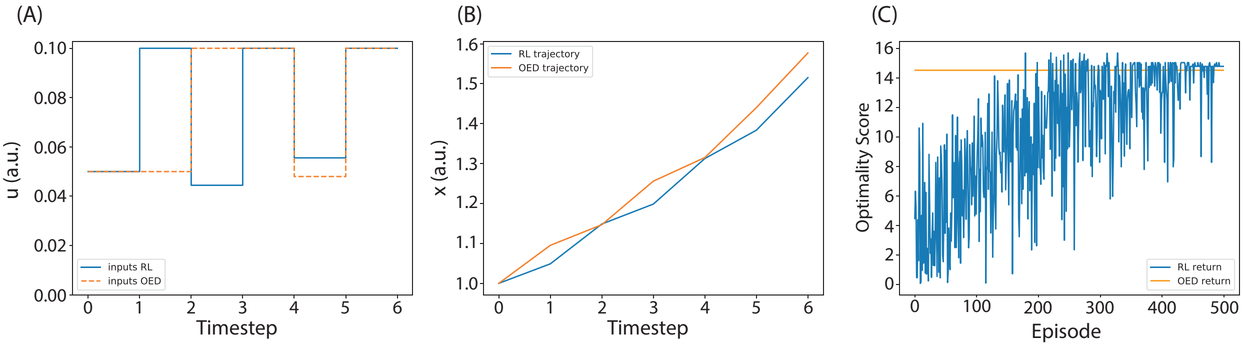

Figure A: Reinforcement learning for optimal experimental design on a simple non-linear system. (A) The input profiles of the two agents. (B) The corresponding state trajectories. (C) The training performance of the RL agent compared to the performance achieved by the OSAO.

2 Full value fitting results

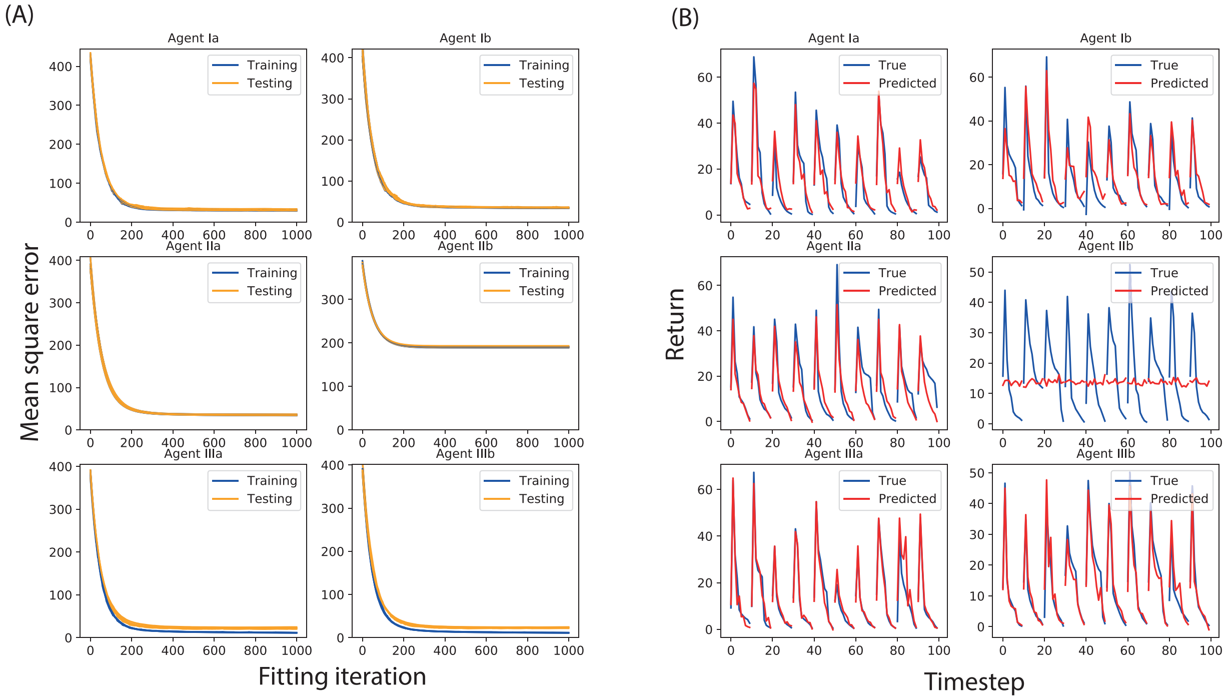

Figure B: Full value fitting results (A) Training and testing performance of all agents over 1000 Fitted Q-iterations. (B) The true vs predicted return of 10 experiments form the testing set for all agents after training.

3 Recurrent FQ

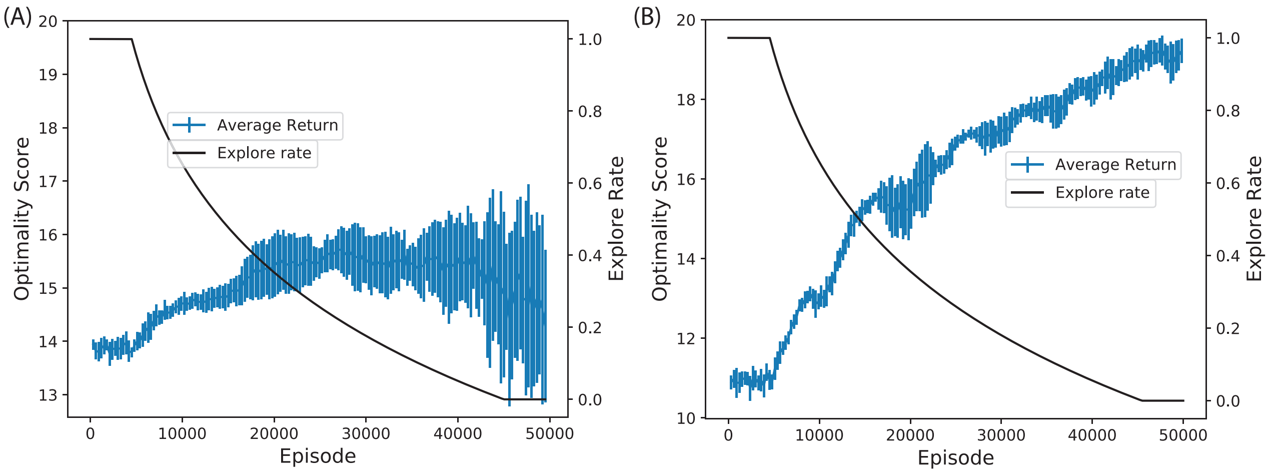

Figure C: Recurrent FQ for optimal experimental design to infer the values of model parameters for an auxotrophic bacterial strain growing in a chemostat. (A) Average training progress of ten recurrent FQ-agents over 50000 episodes where the agents are learning in the full action space of 100 action choices. The return of the FQ-agents is averaged across the 10 repeats. The mean is shown, along with error bars indicating one standard deviation. (B) The same results for fitted Q-learning agents in a smaller actions space of only 4 possible action choices.

4 RT3D performance for different parameter samples

As a comparison to the other methods the error in independent parameter estimates was found (see Methods for details) where the best performing repeat from Fig 4A was used as the RT3D controller. Here the true system parameters were sampled from the uniform distribution before each repeat to assess the ability of the experimental designs to fit the parameters of different parametrisations across the distribution. To remove the dependence of the OSAO and MPC on knowledge of the system parameters they were initialised with the centre of the parameter distribution. Because the system takes different parametrisations for each experiment, we discard the determinant of the covariance matrix of parameters and the optimality score, as these will both be highly dependent on the samples taken, and focus on the normalised MSE in the resulting inferred parameters. Independent parameter samples were taken for each method. These results are shown in Table A, which reveal that in comparison to the previous section the performance of the Rational design, OSAO and MPC have drastically declined. This is expected, because they are ignorant of the changing parametrisations and has no mechanism to adapt their experimental design depending on where in the parameter distribution the true system happens to be. In contrast, the RT3D controller has maintained its performance better, with a smaller increase in the total parameter error. This is further verification that the RT3D controller can adapt the experiment online and that this leads to more informative experiments. The normalised squared error for each parameter sample was plotted on the log scale for each experimental design (Fig D). These were ordered from low to high error for each design. This shows that the error of the T3D controller is consistently below that of the other methods for parameters sampled from the distribution and also highlights that the average error is dominated by a few pathological parameter samples.

	Rational	OSAO	MPC	RT3D
Normalised MSE	1.79	1.31	0.89	0.33

Table A: Mean normalised square parameter error of different methods for optimal experimental design on the single auxotroph model over a parameter distribution

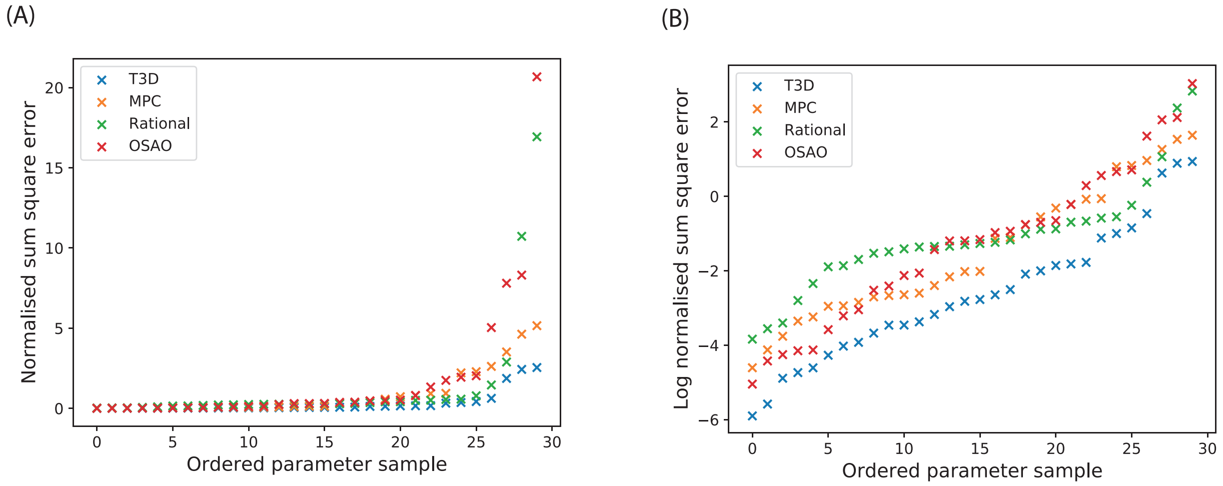

Figure D: Comparison of the error of parameter estimates between different OED methods. The normalised sum square error for each of the 30 parameter samples ordered from lowest to highest for each experimental design (A) and the log normalised sum square error (B)

Parameter value $[\mu_{max}, K_m, K_{m0}]$	MPC	RL
$[0.552564, 0.000400962, 0.0000775143](S1)$	18.85	17.63
$[0.708972, 0.000500437, 0.0000490196](S2)$	20.05	19.03
$[0.500478, 0.00041873, 0.000029529](S3)$	18.02	16.81
$[1.45073, 0.000810734, 0.0000961402](S4)$	21.52	20.41
$[0.5, 0.0001, 0.00001]$ (Lower bound)	18.01	16.78
$[1.25, 0.00055, 0.000055]$ (Centre)	20.79	20.31
$[1, 0.00048776, 0.00006845928]$ (Nominal params)	20.07	20.11
$[2, 0.001, 0.0001]$ (Upper bound)	21.86	20.15

Table B: RT3D controller trained over a parameter distribution compared with an MPC with perfect system knowledge. The optimality score of the experiments produced by each controller is shown for different samples within the distribution.

5 RT3D can design optimal experiments on a model of gene transcription

We implemented the reinforcement learning OED algorithm with the goal of inferring values of the 'intrinsic' parameters of a genetic construct. We distinguish between intrinsic parameters, which govern the gene's induction behaviour, and the physiological parameters which are growth-rate-dependent and reflect the state of the host cell. The model equations [1] are:

$$\frac{d}{dt}\frac{X_{rna}}{V} = \alpha\frac{g}{V}\frac{\frac{P_a}{\eta G}K_r + \frac{P_a K_{rt}}{(\eta G)^2}u(t)}{1 + \frac{P_a}{\eta G}K_r + (\frac{K_t}{\eta G} + \frac{P_a K_{rt}}{(\eta G)^2})u(t)} - \delta\xi\frac{X_{rna}}{V}$$

$$\frac{d}{dt}\frac{X_{prot}}{V} = \frac{\beta\frac{R_f}{V}}{K_M + \frac{R_f}{V}}\frac{X_{rna}}{V} - \lambda\frac{X_{prot}}{V}$$

where X_{rna} and X_{prot} are the concentration of RNA and protein, $\alpha, K_r, K_t, K_{rt}, \delta, \beta$ and K_M are intrinsic parameters, V, g, P_a, G, R_f and λ are growth-rate dependent parameters and η and ξ are fixed. Nominal parameter values are provided in Table C. As control inputs we choose the transcription factor copy number, u. The measurement outputs are the abundance of protein and mRNA: X_{rna} and X_{prot}. We omit K_r from the set of intrinsic parameters to be inferred because it has previously been found to be practically unidentifiable from these output channels [1].

Experiments were designed using rational (human) design, MPC and the RT3D controllers Each experiment lasts 600 minutes. They are divided into six 100-minute long sample-and-hold intervals, each of which is assigned an input value u. The MPC and RT3D methods were allowed to choose each u continuously from within the range $0 < u < 1000$. The RT3D controller outperformed the rational design and performed equivalently to the MPC in terms of the optimality score (Table C).

The covariance of 30 independent parameter estimates was calculated. The resulting logarithm of the determinant of the covariance matrix of the parameters is reported in Table D, from which we see that there is good agreement between the optimality scores and the co-variance in the parameter estimates.

The experimental inputs and resulting trajectories for the three experimental designs are shown in Fig EA-C. The designs from the OSAO and the RL optimisers show similar input profiles, which is expected because they share the same objective. Fig ED shows the training performance of 3 independent RL controllers, of which the best performing one was used to design the experiment in Fig EC. We see that the resulting performance at the end of training is consistently high compared with the rational design and OSAO and approximatley the same as the MPC. These results confirm

Intrinsic parameter	Value	Minimum	Maximum	Unit
α	20	1	30	min^{-1}
K_t	5×10^5	2×10^3	1×10^6	AU
K_{rt}	1.09×10^9	4.02×10^5	5.93×10^{10}	AU
δ	2.57×10^{-4}	7.7×10^{-5}	7.7×10^{-4}	$\mu m^{-3}\text{min}^{-1}$
β	4.0	1	10	min^{-1}
K_M	750	750	1500	μm^{-3}
Physiological parameter	Value			Unit
g	5.7			AU
P_a	4000			AU
G	4.3			AU
η	900			$\mu m^{-3}\text{min}^{-1}$
R_f	7000			AU
V	2.24			μm^{-3}
λ	3.5×10^{-2}			min^{-1}

Table C: The intrinsic parameters of the gene transcription system. Parameter values used for simulations of an inducible gene transcription system and their minimum and maximum bounds when sampling from a distribution.

	Rational	MPC	RT3D
D-optimality	67.73	73.98	73.97
$\log\|cov(\theta)\|$	-33.16	-34.07	-34.13
Normalised MSE	0.014	0.0097	0.0097

Table D: Performance metrics of different methods for experimental designs relating to the gene transcription model.

that our approach of reinforcement learning for OED can produce D-optimal experimental designs for reducing uncertainty in the inference of parameter values for a complex nonlinear biological model.

Finally, we tested the ability of the RT3D agent to learn over a parameter distribution. A uniform distribution defined defined by the ranges in Table C was sampled for each episode. The training performance of ten agents is shown in S Fig E, where we can that the agents have learned to increase the average optimality over the training time and the performance is consistent between the different repeats. The parameter inference ability of the best of these repeats was compared to an MPC controller which designed an experiment using parameters in the centre of the distribution. As expected the results in Table E show that the RL agent outperforms the MPC.

	Rational	MPC	RT3D
Normalised MSE	1.77	0.61	0.36

Table E: Mean normalised square parameter error of different methods for optimal experimental design on the gene transcription model over a parameter distribution

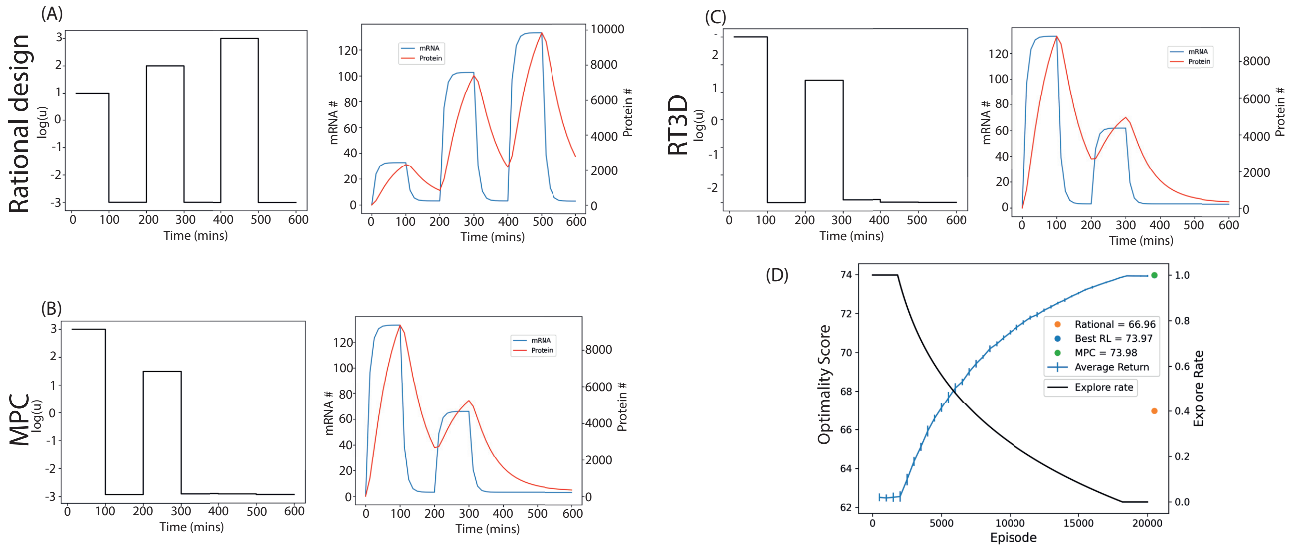

Figure E: Experimental designs for parameter inference for the gene expression model. Experiments produced by rational (human) design (A), one step ahead optimisation (OSOA) (B) and reinforcement learning (RL) (C) with corresponding system trajectories. (D) Average return of ten RT3D agents over 20000 episodes and the scores of the OSOA and rational design. This was then averaged across the 10 repeats.

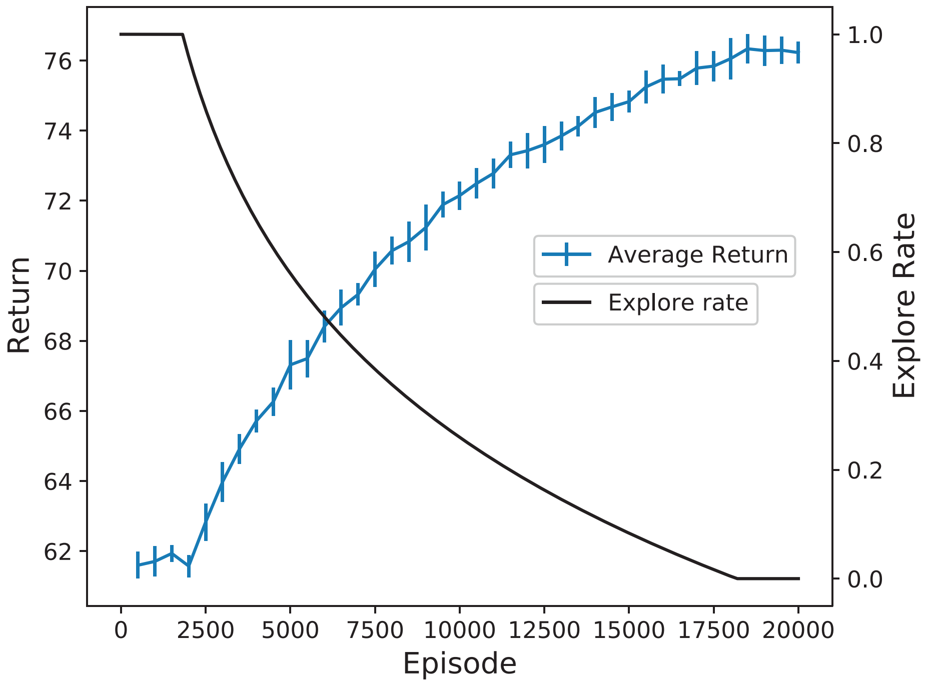

Figure F: The RT3D algorithm trained over a parameter distribution on the gene expression model. . Average return of ten RT3D agents over 20000 episodes. This was then averaged across the 10 repeats.

References

[1] Nathan Braniff, Matthew Scott, and Brian Ingalls. Component characterization in a growth-dependent physiological context: optimal experimental design. *Processes*, 7(1):52, 2019.

[2] Joel A E Andersson, Joris Gillis, Greg Horn, James B Rawlings, and Moritz Diehl. CasADi – A software framework for nonlinear optimization and optimal control. *Mathematical Programming Computation*, 11(1):1–36, 2019.
