# Supplementary material for: Deep reinforcement learning for optimal experimental design in biology
Source: PLoS Comput Biol. 2022 Nov 21;18(11):e1010695. doi: 10.1371/journal.pcbi.1010695 (PMC9721483; doi:10.1371/journal.pcbi.1010695)
Supplement: Attachment — Submitted filename: PLOS-response-to-reviewers.docx [file pcbi.1010695.s002.docx]

**Deep Reinforcement Learning for Optimal Experimental Design in Biology**

**Response to Reviewers**

We would like to thank all of the reviewers for their comments and the time that they have spent reviewing our manuscript. Below we have detailed responses to all of the points raised. The original comments are included in black and our responses given in blue below.

**Reviewer 1**: 

Comment 1: The literature review in the introduction should be updated, especially regarding:
(i) links between OED and control theory (see e.g. surveys by Gevers, or Hjalmarsson; also papers from Pronzato such as <https://doi.org/10.1016/j.automatica.2007.05.016>)

Response: we have now cited some of these works in the introduction.

(ii) other recent related approaches, e.g. <https://arxiv.org/abs/2202.00821>

Response: we have included some citations and consideration of recent approaches in the introduction

Comment 2: previous works (e.g. by Schenkendorf and others) have illustrated the use of global sensitivity analysis in OED to address some of the concerns outlined in this study. Such works should be cited/discussed.

Response: we have added GSA into our discussion of different OED methods in the discussion section, citing and discussing such works.

Comment 3: the authors have focused on D-optimal design criterion. Previous works (also not cited in this study) have shown a trade-off between different FIM-based metrics (e.g. D and mod-E). Maybe a multicriteria approach could be used (as previously done by several groups). It would be nice if the authors discuss this aspect.

Response: we have greatly expanded our consideration of these metrics in the discussion section and how they could be incorporated into our method.

Comment 4: I think the author should also check (and cite/discuss) the excellent survey by Macchietto ( https://doi.org/10.1016/j.ces.2007.11.034 ) which, although not very recent, discusses in detail several of the above issues and the related literature

Response: we agree this review is excellent and we thank the reviewer for bringing it to our attention. We have cited and incorporated ideas from this review throughout the introduction and discussion

Comment 5: the authors show that a deep reinforcement learning approach performs favourably in comparison with a one-step ahead optimisation algorithm and a model predictive controller using a rather simple case study (bacterial growth in a chemostat). How scalable is their approach for more realistic applications?

Response: we have expanded our discussion of this aspect in the discussion section. We have also incorporated some results in the supplementary which demonstrate the use of our method on a gene transcription system which has previously been used in OED research.

Comment 6: also regarding scalability, recent alternative BO-based approaches should also be discussed (e.g. https://doi.org/10.23919/ACC45564.2020.9147824 )

Response: We have expanded the consideration of Bayesian approaches in the discussion to acknowledge this line of work

Minor issues:
- some refs are incomplete

Response: fixed

**Reviewer 2:**

Comment 1: Given the audience of this journal, I believe that the manuscript would greatly benefit from a section that outlines the key elements of Reinforcement Learning and clarifies some of the jargon (Fitted Q, value function etc);

Response: we have added a paragraph in the first results section to explain the key concepts in RL

- While the authors compare their RL agents to MPC and one step ahead optimization I think that it would be helpful to show how the solutions the best agent identifies compares to other optimisation schemes commonly used in dynamic experimental design (e.g. eSS). Also, connected, RL is presented as a solution but the rationale for using ML in the first place is not entirely clear.

Response: we have added some rational and previous work for the use of ML in OED in the introduction. We agree that it will be fruitful to more fully compare the method to other optimisation schemes. However, we believe this is better left for future work when a more thorough comparison can be made. In this initial work we have compared against an MPC with perfect system knowledge (which is unfair on our method) and we believe this is suitable to demonstrate that our methodology has potential for future research.

- In Section 4.4 I found the speculation on the reason for the underperformance ("problem too complex") requiring a level of suspension of judgement that I was not entirely comfortable with. Could the authors simplify the problem (less control intervals or less discrete values of the inputs) to show that "problem complexity" is indeed the issue?

Response: we agree that the suggested demonstration would strengthen our rational here and we have run a simplified version of the control problem using a smaller action space and demonstrated that the agents learn well. (SFig 3B)

- At page 5, where the chemotast model is introduced, the authors should review the vay the parameters are introduced as it seems inconsistent with the text that explains them;

Response: fixed

- The manuscript is sprinkled with a parentheticals, some dispensable (e.g. I don't think this audience needs to be told what an auxotroph is). I would encourage the authors to go over the text and ask, for each sentence between parentheses, whether it is necessary. If so, make a proper sentence of it, otherwise eliminate;

Response: we have gone through and removed almost all of these parenthesis as suggested.

- At page 11: "the determinant of the Fisher Information" should read "the determinant of the Fisher Information matrix"

Response: fixed

Overall I genuinely believe this is an excellent piece of work and the authors should be commended for their contribution.
