# Supplementary material for: Deep reinforcement learning for optimal experimental design in biology
Source: PLoS Comput Biol. 2022 Nov 21;18(11):e1010695. doi: 10.1371/journal.pcbi.1010695 (PMC9721483; doi:10.1371/journal.pcbi.1010695)
Supplement: Attachment — Submitted filename: PLOS-response-to-reviewers.docx [file pcbi.1010695.s003.docx]

**Deep Reinforcement Learning for Optimal Experimental Design in Biology**

**Response to Reviewers**

We would like to thank the reviewer for their comments and the time that they have spent reviewing our manuscript. Below we have detailed responses to all of the points raised. The original comments are included in black and our responses given in blue below.

**Reviewer 1**: 

ref 10 must be corrected (it shows the funding info, not the journal)

- Fixed

the annotated coloured version is badly compiled regarding refs etc., but I was able to see the changes anyway

- We have ensured the above changes to the reference are highlighted
